# Proton and Oxide Ion Conductivity in Palmierite Oxides

**DOI:** 10.1021/acs.chemmater.2c01218

**Published:** 2022-09-06

**Authors:** Sacha Fop, James A. Dawson, Dylan N. Tawse, Matthew G. Skellern, Janet M. S. Skakle, Abbie C. Mclaughlin

**Affiliations:** † ISIS Facility, Rutherford Appleton Laboratory, Harwell OX11 0QX, United Kingdom; ‡ The Chemistry Department, University of Aberdeen, Aberdeen AB24 3UE, United Kingdom; § Chemistry − School of Natural and Environmental Science, Newcastle University, Newcastle NE1 7RU, United Kingdom; ∥ Centre for Energy, Newcastle University, Newcastle NE1 7RU, United Kingdom

## Abstract

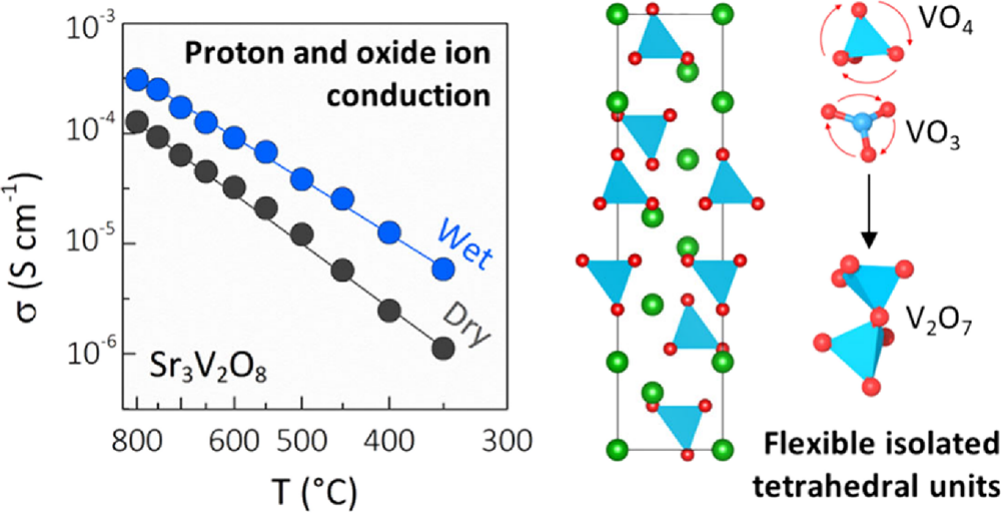

Solid proton and oxide ion conductors have key applications in several hydrogen-based and energy-related technologies. Here, we report on the discovery of significant proton and oxide ion conductivity in palmierite oxides A_3_V_2_O_8_ (A = Sr, Ba), which crystallize with a framework of isolated tetrahedral VO_4_ units. We show that these systems present prevalent ionic conduction, with a large protonic component under humidified air (*t*_H_ ∼ 0.6–0.8) and high protonic mobility. In particular, the proton conductivity of Sr_3_V_2_O_8_ is 1.0 × 10^–4^ S cm^–1^ at 600 °C, competitive with the best proton conductors constituted by isolated tetrahedral units. Simulations show that the three-dimensional ionic transport is vacancy-driven and facilitated by rotational motion of the VO_4_ units, which can stabilize oxygen defects via formation of V_2_O_7_ dimers. Our findings demonstrate that palmierite oxides are a new promising class of ionic conductors where stabilization of parallel vacancy and interstitial defects can enable high ionic conductivity.

## Introduction

Solid proton and oxide ion conductors are important materials with applications in a range of hydrogen-based electrochemical energy technologies, from ceramic fuel cells (CFCs) to electrolyzers (SOECs) and chemical reactors.^1−9^ To make these technologies more economical and durable, it is important to develop ionic conductors with high conductivities at reduced temperatures (≤ 600 °C). Ionic conduction strongly depends on the characteristics of the crystal structure, and the discovery of new solid proton and oxide ion conductors crucially depends on exploring novel structure types and materials.

Several oxide ion and proton conductors crystallize with the perovskite ABO_3_ structure, which is composed by a three-dimensional network of corner-sharing BO_6_ octahedra.^10−14^ In these perovskite-type materials, the defects necessary for ionic transport are usually created with the insertion of extrinsic oxygen vacancies by acceptor doping.^10,14,15^ The oxygen vacancies can enable oxide ion diffusion and provide sites for the dissociative absorption of water and creation of protonic defects. Oxide ion migration in perovskite-type oxides occurs via vacancy hopping between oxygen sites along a BO_6_ octahedron edge,^15,16^ whereas proton transport generally follows a Grotthuss mechanism characterized by the fast rotational diffusion of the protonic defect around an oxygen atom followed by intra-octahedral hopping toward a neighboring oxide ion.^10,17^ Proton and oxide ion diffusion along frameworks of corner-sharing octahedral units is a common feature of several ionically conducting oxides.^18−20^ Reports of significant proton or oxide ion transport in oxide structures constituted by isolated tetrahedral units are on the other hand relatively scarce. Examples comprise proton conduction in acceptor-doped scheelite- and monazite-type oxides LaMO_4_ (M = P, V, As, Nb, Sb, Ta),^21−26^ lanthanum–barium gallates of general formula La_1-*x*_Ba_1+*x*_GaO_4-*x*/2_,^27,28^ and acceptor-doped Gd_3_GaO_6_.^29^ Similarly, oxide ion conduction has been reported only in a few structural families constituted by isolated tetrahedral units, namely, apatites and scheelites.^30−32^

**Figure 1:**
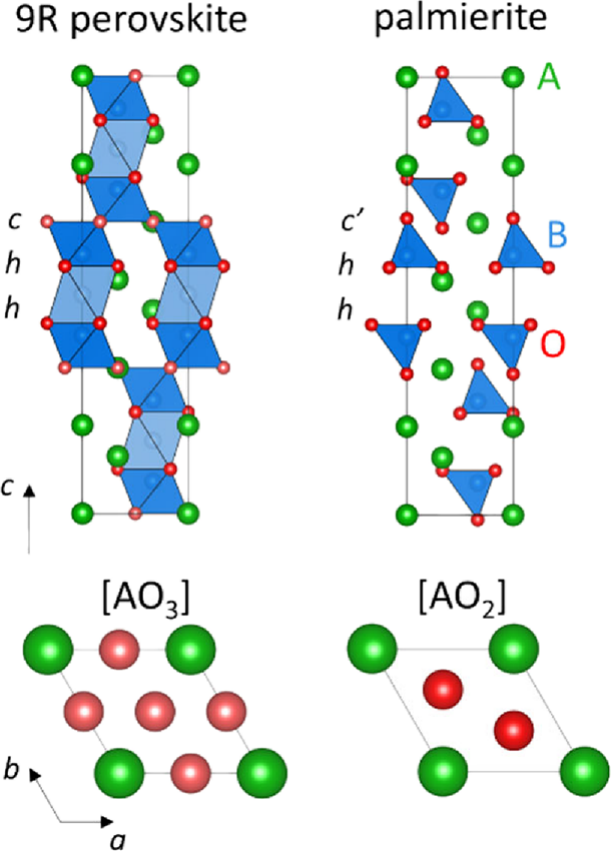
Comparison between the 9R hexagonal perovskite and palmierite structures. The palmierite structure is a cation-deficient derivative of the 9R perovskite where the cubic [AO_3_] layer of the stacking sequence (hhc)_3_ is replaced by an oxygen-deficient [AO_2_] layer (c′). Crystallographically, the palmierite structure can be obtained from the 9R perovskite by the shifting of the oxygen atom on Wyckoff position 9*e* (1/2,0,0,0) to 6*c* (0,0,∼ 1/3). Note how the central octahedron of the metal trimers is unoccupied in the palmierite structure.

We have recently reported proton and oxide ion conduction in a series of cation-deficient hexagonal perovskite derivatives formed by a disordered combination of perovskite and palmierite-like layers.^33−40^ The latter are composed by isolated tetrahedral units that have a particular topology that allows water incorporation and fast ionic transport. This is demonstrated by the high proton and oxide ion conductivity exhibited by the hexagonal perovskite derivative Ba_7_Nb_4_MoO_20_ (respectively 4.0 × 10^–3^ and 2.0 × 10^–3^ S cm^–1^ at 500 °C) and the high oxide ion conductivity of Ba_3_Nb_0.9_V_0.1_MoO_8.5_ (1.0 × 10^–2^ S cm^–1^ at 600 °C), both comparable to state-of-the-art doped perovskite-type ionic conductors.^39,40^ On the basis of such findings, we have investigated the ionic conductivity of palmierite oxides with composition A_3_V_2_O_8_ (A = Sr, Ba). The palmierite structure is a cation-deficient derivative of the 9R hexagonal perovskite polytype A_3_B_2_O_9_, where the cubic [AO_3_] layer of the stacking sequence (hhc)_3_ is replaced by a layer of composition [AO_2_], thus generating layers of isolated tetrahedral units spaced by empty octahedral sites (Figure 1).^41,42^ Here, we report on the discovery of significant proton and oxide ion conductivity in A_3_V_2_O_8_ for the first time, thus demonstrating that palmierite oxides with isolated tetrahedral units can constitute a new family of ionic conductors.

## Experimental Section

### Synthesis and Characterization

Samples of A_3_V_2_O_8_ (with A = Sr, Ba) were synthesized by the solid-state reaction method. Stoichiometric amounts of SrCO_3_ (99.995%, Aldrich) or BaCO_3_ (99.999%, Aldrich) and V_2_O_5_ (99.95%, Aldrich) were ground, pressed into a pellet, heated at 1100 °C for 10 h, and then cooled to room temperature at 5 °C/min. The heating step was repeated a second time to obtain a phase-pure product. Sample purity was confirmed by laboratory X-ray diffraction (XRD) using a PANalytical Empyrean diffractometer equipped with a Cu K_α_ tube and a Johansson monochromator. Data were recorded in the range 10° < 2θ < 120° with a step size of 0.013°. SEM-EDS analysis was performed using a field-emission gun Carl Zeiss Gemini SEM 300 equipped with an AZtec Energy EDS analysis system with an XMax 80 detector and an AZtecHKL EBSD analysis system with a Nordlys Nano EBSD camera (Oxford Instruments Ltd.). SEM micrographs were collected on the surface and section of carbon-coated sintered dense pellets of A_3_V_2_O_8_ (A = Sr, Ba). Energy-dispersive spectroscopy (EDS) analysis performed on different areas of the pellets indicated cation compositions of Sr:V = 3.01(3):2.00(1) and Ba:V = 3.09(5):1.95(4), in agreement with the nominal compositions. Thermogravimetric analysis (TGA) was performed with a Mettler Toledo TGA 2 on samples stored at ambient conditions. The samples were heated at 200 °C for 10 h prior to the analysis in order to remove any adsorbed surface water. Thermogravimetric measurements of water uptake were carried out by recording the weight increase of the dried samples upon cooling in equilibrium isotherms under humidified air (*p*H_2_O ≈ 0.021 atm), with 2 h stabilization time at each temperature step.

### Impedance Spectroscopy

Dense pellets (∼ 90% of the theoretical neutron diffraction density) of approximately ∼ 1 mm thickness and ∼ 10 mm diameter were prepared by sintering samples at 1100 °C for 2 h and used for the electrical characterization after application of platinum electrodes. The electrical properties of A_3_V_2_O_8_ (A = Sr, Ba) were measured by AC impedance spectroscopy with a Solartron 1260 impedance analyzer in the frequency range 0.1 Hz–1 MHz, applying an alternating voltage of 0.1 V. Measurements were taken upon cooling the samples from 800 °C under a range of atmospheres in a sealed tube furnace and allowing 2 h of equilibration at each temperature step. For the measurements in air, O_2_, N_2_, and 5% H_2_/N_2_, the employed gas was dried by flowing through a column of a commercial desiccant (Drierite) (*p*H_2_O < 10^–4^ atm). Humidified air was produced by bubbling air through a water-filled Dreschel bottle at ambient temperature (*p*H_2_O ∼ 0.021 atm). Total resistivity values (*R*_b_ + *R*_gb_) were extracted from the high-frequency intercept of the arcs on the real impedance axis. In addition, equivalent circuit analysis was used to extract the individual bulk and grain boundary responses for Sr_3_V_2_O_8_; a detailed description of the analysis can be found in the Supporting Information.

### Neutron Diffraction and Structural Analysis

Room-temperature high-resolution neutron diffraction experiments were performed on the Time-of-Flight (TOF) High-Resolution Powder Diffractometer (HRPD) at ISIS (Rutherford Appleton Laboratory, Harwell, Oxford, UK). Five grams of A_3_V_2_O_8_ (A = Sr, Ba) powder samples were loaded into a vanadium can and measured at room temperature (25 °C) with a total scan time of 4 h.

Rietveld analysis was performed using the GSAS/EXPGUI package.^43^ The palmierite structure reported in ref (42) was employed as the initial model for the Rietveld refinements. The metal A cations are on two different positions, A1 (at Wyckoff site 3*a*) and A2 (at Wyckoff site 6*c*), while the vanadium atoms occupy a single position (at Wyckoff site 6*c*). The oxygen atoms are on two positions, O1 at Wyckoff site 6*c* and O2 at Wyckoff site 18*h*. Data from both the high-resolution back-scattering detector bank and the 90° detector bank were employed for the structural refinements of the measurements collected on HRPD. The background was fitted with the Chebyshev polynomial function, and peak shapes were modeled using a pseudo-Voigt function.

The refined structural models were employed as input for the bond-valence site energy (BVSE) calculations with the softBV program.^44,45^ Migration pathways and barriers for test H^+^ and O^2–^ ions were calculated for a dense grid of points with a resolution of 0.1 Å. The calculated energy profiles and isosurfaces were employed to identify regions of low bond-valence site energy corresponding to energy minimum equilibrium sites and diffusion pathways.

### Computational Methods

The Vienna ab initio simulation package (VASP) was used to carry out density functional theory simulations. A plane-wave cutoff energy of 520 eV and a *k*-point mesh spacing smaller than 0.05 Å^–1^ were utilized for the geometry optimization calculations. All calculations were performed by employing the projector augmented wave method^46^ and the PBEsol exchange-correlation functional.^47^ Single unit cells and 2 × 2 × 1 supercells of Ba_3_V_2_O_8_ and Sr_3_V_2_O_8_ were used to calculate the hydration energetics, with the number of water molecules (*n*H_2_O) per formula unit equal to 0 (dehydrated), 0.0825, or 0.330. Table S1 reports the unit cell parameters for the computed structures. The energetically preferred sites for the additional oxygen ions and protons were determined using systematic static DFT calculations. The oxygen and hydroxyl sites were found by placing them at all possible symmetrically inequivalent sites and calculating the resulting energies. The initial position of each proton was determined by first attaching it to the relevant oxygen ion and then systematically rotating it around this ion to find the lowest energy position, i.e., its preferred site. Ab initio molecular dynamics (AIMD) simulations were performed with a plane-wave cutoff energy of 400 eV and the *k*-space was sampled using the gamma-point only. To investigate proton transport, AIMD runs of 50 ps at 800, 1000, and 1200 K were used in 2 × 2 × 1 A_3_V_2_O_8_·0.0825H_2_O and A_3_V_2_O_8_·0.330H_2_O (A = Sr, Ba) supercells respectively containing 168 and 159 ions using the canonical NVT ensemble with the Nose–Hoover thermostat.^48^ A time step of 1 fs was used to account for the motion of the protons. To determine the oxide ion transport mechanism, 30 ps AIMD simulations of Sr_3_V_2_O_8_ with and without oxygen vacancies at 1200 K were carried out using a 2 × 2 × 1 supercell and a time step of 2 fs. The selection of oxygen vacancy sites was simplified by the fact that the energy differences between the two oxygen sites (6*c* and 18*h*) in Ba_3_V_2_O_8_ and Sr_3_V_2_O_8_ are minimal (<0.06 eV).

## Results and Discussion

### Ionic Conductivity

**Figure 2:**
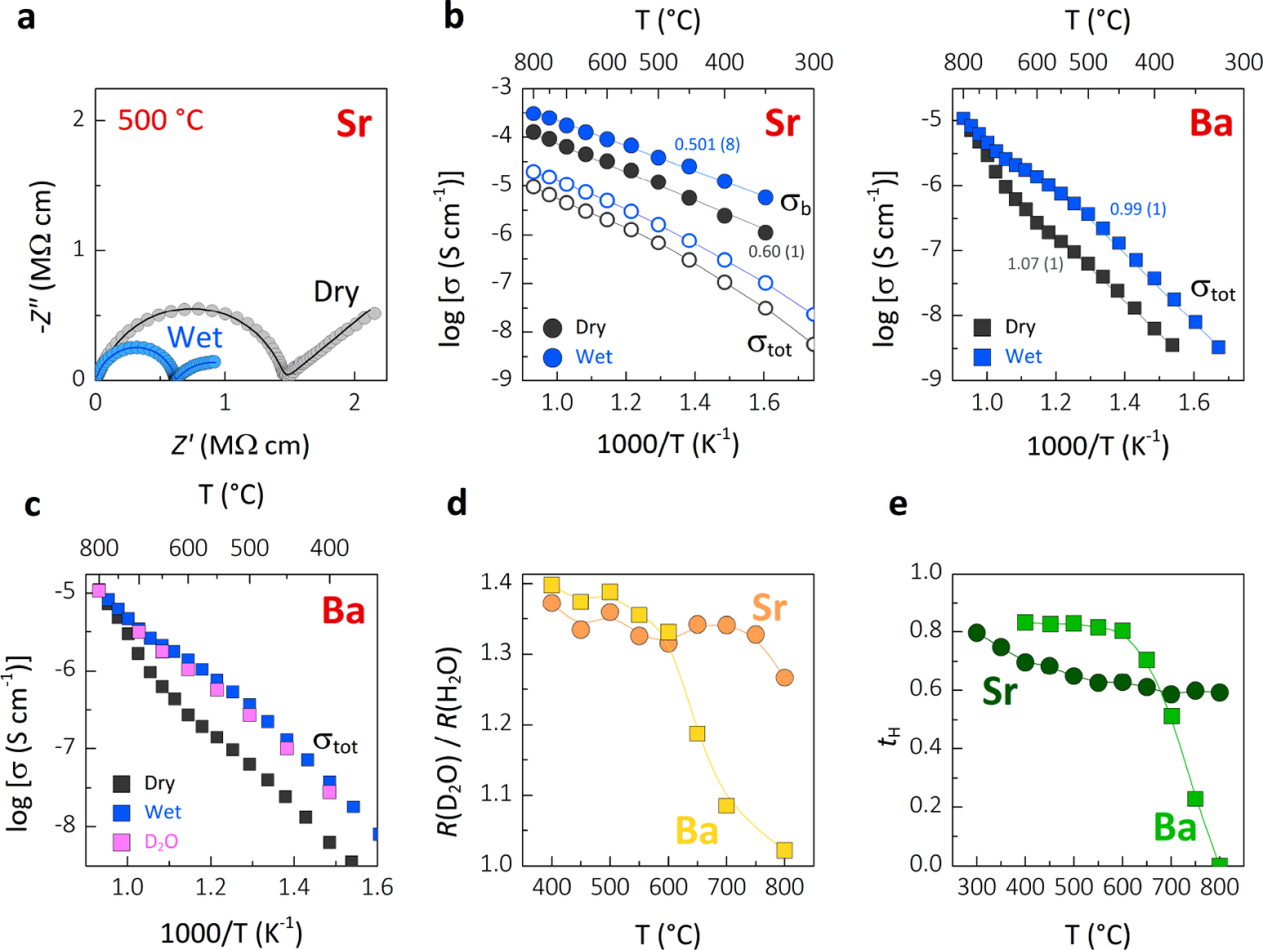
(a) Typical complex impedance plots for Sr_3_V_2_O_8_ collected under dry and humidified air. The line represents the equivalent circuit fitting to the data. (b) Arrhenius plots of the bulk and total conductivity of Sr_3_V_2_O_8_ and the total conductivity of Ba_3_V_2_O_8_ collected under dry and humidified air atmospheres with the respective activation energies (in eV). (c) Arrhenius plot showing a reduction of the total conductivity of Ba_3_V_2_O_8_ under air + D_2_O atmosphere due to the isotope effect. (d) Plot of the resistivity ratio of the sample in air + D_2_O and air + H_2_O. (e) Calculated proton transport number.

A_3_V_2_O_8_ samples were synthesized via solid-state reaction at 1100 °C. The purity of the as prepared phases was confirmed by laboratory X-ray diffraction (Figure S1). X-ray diffraction patterns collected after annealing the samples at different temperatures in a range of atmospheres (O_2_, 5% H_2_/N_2_, and humidified air) demonstrated the phase stability of the A_3_V_2_O_8_ materials (Figure S2). AC impedance spectroscopy measurements under variable atmospheres were employed to investigate the ionic conductivity of A_3_V_2_O_8_. Measurements were performed on dense pellets (∼90% of the theoretical neutron diffraction density) as confirmed by SEM micrographs (Figure S3). Typical complex impedance Z* plots recorded in dry (*p*H_2_O < 10^–4^ atm) and humidified (*p*H_2_O ∼ 0.021 atm) air are shown in Figure 2a and Figure S4. The plots for Sr_3_V_2_O_8_ show a broad and depressed grain arc constituted by the overlap of the bulk (∼ 6.2–8.3 pF cm^–1^) and grain boundary (∼ 0.01–0.02 nF cm^–1^) responses, which are clearly identifiable in the complex modulus (*M*″) plots (Figure S5a,b).^49^ The data of Ba_3_V_2_O_8_ show a single signal, which can be associated to the overall grain response (∼ 4.7–6.5 pF cm^–1^) (Figure S5c,d). The complex impedance plots display a clear reduction in resistivity under humidified air. The Arrhenius plots in Figure 2b show a marked increase in conductivity, indicating proton conduction. The bulk conductivity of Sr_3_V_2_O_8_ in humidified air (1.0 × 10^–4^ S cm^–1^ at 600 °C) is higher than the bulk conductivity measured in dry air (3.2 × 10^–5^ S cm^–1^). Ba_3_V_2_O_8_ shows an increase in conductivity of about one order of magnitude in humidified air, from 1.8 × 10^–7^ to 1.6 × 10^–6^ S cm^–1^ at 600 °C. Impedance spectroscopy measurements in air humidified with D_2_O clearly show a reduction in conductivity due to the isotope effect, thus further confirming the presence of proton conduction (Figure 2c and Figure S8). The ratio between the resistivity values measured in air + D_2_O and air + H_2_O is 1.3–1.4 (Figure 2d), approaching the expected theoretical value of .^50^ The total conductivity values measured in dry and humidified air atmospheres were employed to calculate the proton transport number, *t*_H_, which corresponds to the ratio of proton conductivity to total conductivity.^51^*t*_H_ is in the range of ∼ 0.6–0.8 for Sr_3_V_2_O_8_ and ∼ 0.82 below 600 °C for Ba_3_V_2_O_8_, demonstrating significant proton conductivity (Figure 2e).

The presence of a Warburg electrode signal in the low frequency part of the complex impedance plots both in dry and humidified atmospheres (Figure 2a and Figure S6) is distinctive of ionic conduction.^49^ In particular, the prominent Warburg spike in dry air atmosphere would indicate oxide ion conduction. Further measurements under a range of dry atmospheres (air, O_2_, N_2_, and 5% H_2_ in N_2_) demonstrate that the total conductivity is independent of oxygen partial pressure (Figure S9), confirming that A_3_V_2_O_8_ is predominantly an oxide ion conductor at high and intermediate *p*O_2_ values. The increase in conductivity under the 5% H_2_ in N_2_ atmosphere would suggest an electronic *n*-type component. However, the electronic component is small, and the conductivity is predominantly ionic, as confirmed by the presence of clear electrode signals at all temperatures under the more reducing conditions (Figure S9a), which would have been absent in case of largely electronic transport.^52^

**Figure 3:**
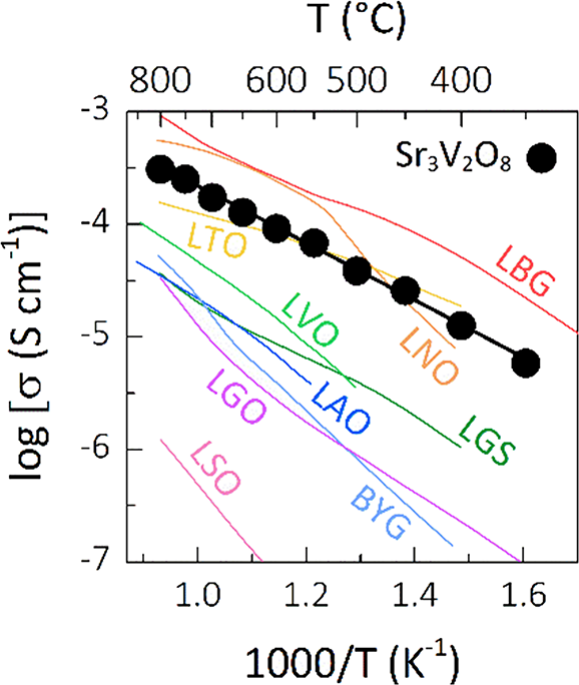
Arrhenius plot of the bulk proton conductivity of Sr_3_V_2_O_8_ in humidified air compared with the proton conductivities of La_0.8_Ba_1.2_GaO_3.9_ (LBG),^27^ La_0.99_Ca_0.01_NbO_4_ (LNO),^22^ La_0.99_Ca_0.01_TaO_4_ (LTO),^23^ La_0.99_Ca_0.01_VO_4_ (LVO),^24^ La_0.99_Sr_0.01_AsO_4_ (LAO),^25^ La_0.99_Ca_0.01_SbO_4_ (LSO),^26^ Ba_3_YGa_2_O_7.5_ (BYG),^53^ La_3_Ga_5.06_Si_0.94_O_14_ (LGS),^54^ and La_4_GaTiO_9.5_ (LGO).^55^

These results demonstrate for the first time that A_3_V_2_O_8_ presents significant proton and oxide ion conduction, with the A = Sr sample showing the highest conductivity. In particular, the bulk proton conductivity of palmierite Sr_3_V_2_O_8_ is competitive with the conductivities of acceptor-doped La_0.99_Ca_0.01_MO_4_ (M = Nb, Ta)^22,23^ and La_0.8_Ba_1.2_GaO_3.9_,^27^ which are among the best proton conductors constituted by isolated tetrahedral units. The conductivity of Sr_3_V_2_O_8_ is also significantly higher than the proton conductivities of the oxygen-deficient perovskite derivative Ba_3_YGa_2_O_7.5_^53^ and of doped langasite La_3_Ga_5_SiO_14_,^54^ both containing tetrahedral units with only three of their four corners connected to other framework polyhedra, and of cuspidine La_4_Ga_2_O_9_-based oxides,^55^ which are formed by corner-sharing tetrahedral units (Figure 3). We anticipate that targeted chemical doping will offer a viable route to further enhance the proton and oxide ion conductivity in these undoped A_3_V_2_O_8_ palmierite oxides.

### Crystal Structure

**Figure 4:**
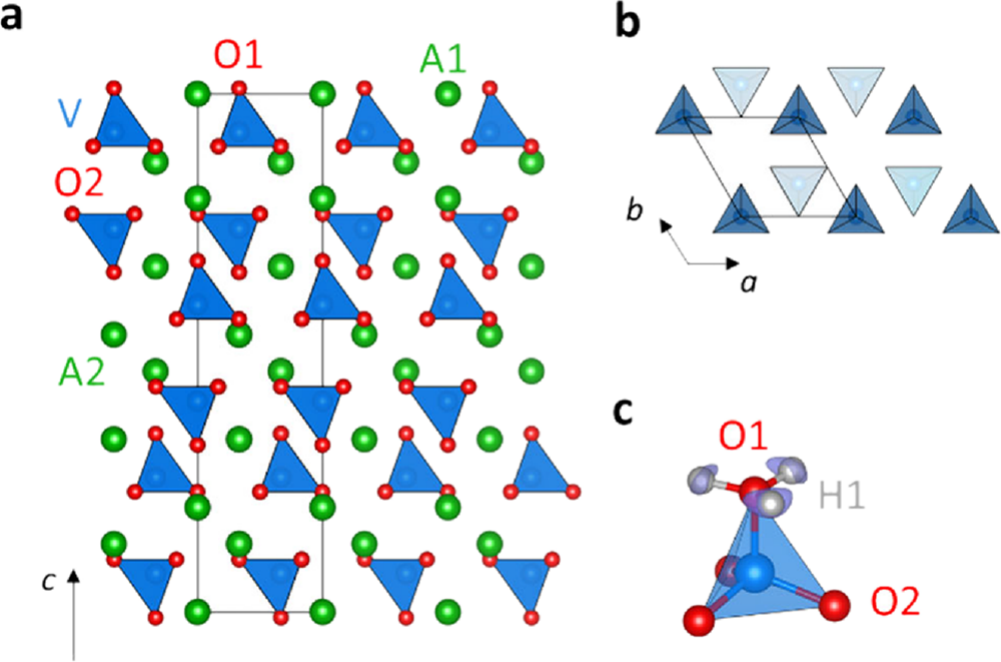
(a) Crystal structure of A_3_V_2_O_8_ (A = Sr, Ba). (b) Distribution of the VO_4_ units within two adjacent tetrahedral layers as seen along the [001] direction. (c) Proton equilibrium position in relation to the VO_4_ tetrahedron as obtained by BVSE and DFT calculations. BVSE isosurface levels are at 0.0 eV.

The structures of A_3_V_2_O_8_ (A = Sr, Ba) were investigated by Rietveld refinement employing the previously reported model.^42^ Preliminary refinement of the crystal structures from high-resolution X-ray diffraction (see Figure S10 and Table S2) showed that A_3_V_2_O_8_ crystallizes with the palmierite structure (space group *R*3̅*m*), which is composed by layers of isolated VO_4_ tetrahedral units spaced by empty cationic vacancies. X-ray difference Fourier maps confirmed that V occupies only the 6*c* Wyckoff site and that the vacancies are ordered (Figure S11), in contrast with what was seen in some hybrid hexagonal perovskite–palmierite Ba_3_*M*′*M*″O_8.5_ (*M*′ = Nb; *M*″ = Mo, W) materials, where the cation vacancies are disordered.^38,39,56,57^ Previous neutron diffraction studies have shown that materials reported to have the palmierite structure can have more complex oxygen ordering, leading to the formation of hexagonal polytype structures with face- or corner-sharing octahedral units, e.g., Ba_3_Nb_2_O_8_.^58^ For this reason, neutron diffraction, which is more sensitive to the oxygen sublattice, was performed on the High-Resolution Powder Diffractometer (HRPD) at the ISIS Neutron and Muon Source at room temperature.^59^ Refinement of the crystal structures from neutron diffraction data resulted in an excellent fit to the data (see Figure S12 and Table S3), thus confirming that A_3_V_2_O_8_ (A = Sr, Ba) indeed crystallize in the palmierite structure with layers of isolated tetrahedral units (Figure 4a,b).

To investigate the possible proton positions, the energy landscape for a test H^+^ was calculated by the bond-valence sum (BVS) method with the software softBV^44,45^ using the structural models from Rietveld refinement as input. The lowest energy (0.0 eV, absolute BVSE minimum) proton site H1 was identified around the apical tetrahedral oxygen O1 at Wyckoff position 18*h* (∼0.11, ∼0.22, and ∼0.32) (Figure 4c). This was also confirmed by DFT calculations for both Sr_3_V_2_O_8_ and Ba_3_V_2_O_8_, with protons either occupying Wyckoff position 18*h* or a site between adjacent oxide ions that acts as a transition state for proton migration. The O1–H1 bond length is ∼ 1.09 and ∼ 1.02 Å from BVS calculations and DFT simulations, respectively, in agreement with values determined in other proton conductors.^60,61^

### Ionic Conduction Mechanism

**Figure 5:**
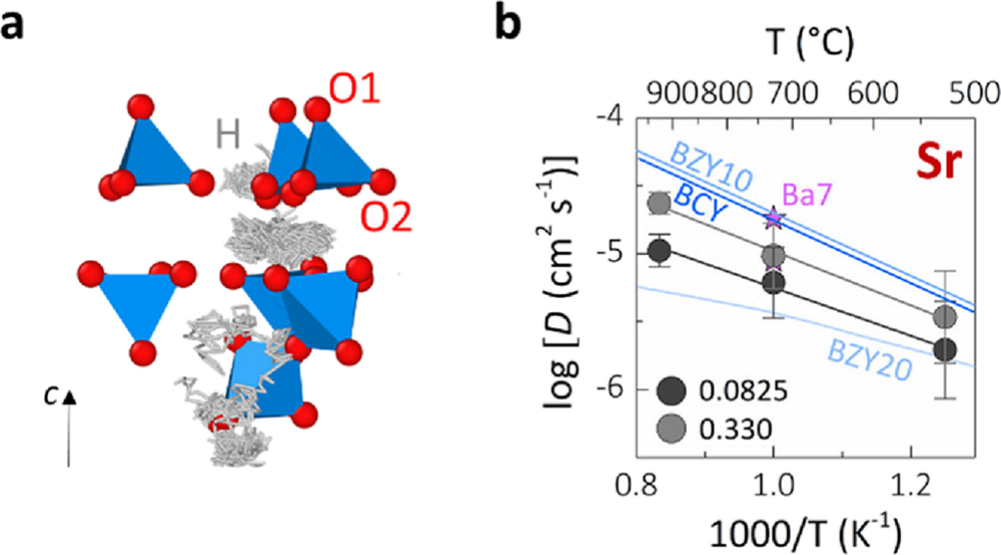
(a) AIMD trajectory plot for protons in Sr_3_V_2_O_8_·0.0825H_2_O at 1200 K. (b) Arrhenius plot of the calculated proton diffusion coefficient for Sr_3_V_2_O_8_·*n*H_2_O, with *n* = 0.0825 and 0.330. The diffusion coefficients for perovskite-type proton conductors BaCe_0.98_Y_0.02_O_3-δ_ and BaZr_0.9_Y_0.1_O_3-δ_ (BCY and BZY10 respectively, both calculated)^62^ and BaZr_0.8_Y_0.2_O_3-δ_ (BZY20, experimental),^63^ as well as for Ba_7_Nb_4_MoO_20_·*n*H_2_O with *n* = 0.125 and 0.5 (Ba7, calculated)^64^ are also plotted for comparison. The error bars correspond to the statistical uncertainty in the fitting of the mean square displacement to time curve.

AIMD simulations at a range of temperatures (800, 1000, and 1200 K) on two different hydrated compositions (A_3_V_2_O_8_·0.0825H_2_O and A_3_V_2_O_8_·0.330H_2_O) were used to further investigate the level and mechanism of proton conduction in these palmierite materials. The AIMD trajectory plot for protons in Sr_3_V_2_O_8_·0.0825H_2_O at 1200 K in Figure 5a shows a three-dimensional percolation network, with long-range proton rotation and hopping primarily along the *ab* plane. Proton hopping occurs across the O1 anions as well as from O1 to O2, but the protons can become trapped by the intrinsic cationic vacancies. BVSE calculations for the interaction of a probe H^+^ ion confirmed the lowest-energy two-dimensional proton conduction pathway, with proton exchange between the H1 positions (BVSE barrier ∼ 0.20 eV) and hopping onto an adjacent O1 atom (BVSE barrier ∼ 0.20–0.23 eV) (Figure S13). While the 2D proton conduction pathway dominates in both Sr_3_V_2_O_8_·0.0825H_2_O and Ba_3_V_2_O_8_·0.0825H_2_O, the AIMD simulations for the materials with a higher water content (A_3_V_2_O_8_·0.330H_2_O) show a transition toward a more isotropic 3D hopping mechanism. To quantify the levels of proton transport in these materials, we calculated the mean squared displacements of the protons and used them to derive proton diffusion coefficients. The calculated diffusion coefficients are presented through Arrhenius plots in Figure 5b and Figure S14. We obtained proton diffusion coefficients of 1.96 × 10^–6^ and 3.42 × 10^–6^ cm^2^ s^–1^ at 800 K for Sr_3_V_2_O_8_·0.0825H_2_O and Sr_3_V_2_O_8_·0.330H_2_O, respectively. In agreement with our conductivity analysis, lower values of 7.89 × 10^–7^ and 1.47 × 10^–6^ cm^2^ s^–1^ at 800 K were found for Ba_3_V_2_O_8_·0.0825H_2_O and Ba_3_V_2_O_8_·0.330H_2_O, respectively. These values are comparable with other high-performance proton conductors^10,62,63^ and the hexagonal perovskite derivative Ba_7_Nb_4_MoO_20_,^64^ which also contains palmierite-like layers, thus confirming the high mobility of the protonic defects in the A_3_V_2_O_8_ structure. These diffusion coefficients were also converted to ionic conductivities using the Nernst–Einstein equation to enable a comparison with our measured conductivities. For Sr_3_V_2_O_8_·0.0825H_2_O and Sr_3_V_2_O_8_·0.330H_2_O, we obtained proton conductivities of 4.61 × 10^–4^ and 8.04 × 10^–4^ S cm^–1^, respectively, at 800 K. These values are in reasonable agreement with the value of 1 × 10^–4^ S cm^–1^ from impedance measurements at 873 K. In contrast, the values for Ba_3_V_2_O_8_·0.0825H_2_O and Ba_3_V_2_O_8_·0.330H_2_O at 800 K were 2.04 × 10^–4^ and 3.79 × 10^–4^ S cm^–1^, respectively, far higher than the experimentally determined value of 1.6 × 10^–6^ S cm^–1^.

Thermogravimetric analysis (TGA) demonstrates that A_3_V_2_O_8_·*n*H_2_O samples stored at ambient conditions have water concentrations of *n* = 0.008 for A = Sr and *n* = 0.013 for Ba (Figure S15a). Thermogravimetric measurements under flow of humidifed air show water uptakes of 0.024 molecules of water per formula unit for Sr_3_V_2_O_8_ and 0.026 for Ba_3_V_2_O_8_ (Figure S15b). These water concentrations are considerably lower than the value of ∼ 0.80 H_2_O molecules per formula unit found for Ba_7_Nb_4_MoO_20_.^40,64^ The low concentrations of water measured in the samples are commensurate with our DFT calculations that predict endothermic hydration enthalpies in the range of 10.61 to 87.80 kJ mol^–1^ (Table S5). These large positive values illustrate the weak hygroscopicity of the materials, particularly when compared to the typically strong exothermic values found for other well-known solid-state proton conductors, including those with palmierite-like layers, such as Ba_7_Nb_4_MoO_20_.^64^ In our previous study of Ba_7_Nb_4_MoO_20_, we found that water is absorbed on the intrinsic oxygen vacancies present on the palmierite-like layer. The hydration leads to a change in the local coordination of the (Nb/Mo)O_*x*_ units and is linked to a shift of the tetrahedral Nb/Mo cation in the palmierite-like layer toward a vacant octahedral site, thus forming (Nb/Mo)O_6_, and results in a strong increase in the exothermic hydration enthalpies (Figure S16a).^64^ To check if A_3_V_2_O_8_ palmierite oxides also present a similar mechanism of water absorption, we performed DFT geometry optimization calculations with a VO_4_ unit changed to a VO_5_/VO_6_ unit as well as with a proton located in a close vicinity to a vanadium ion in an attempt to “push” it from the tetrahedral to octahedral coordination. Results from DFT calculations clearly demonstrate that this phenomenon does not occur in these palmierite oxides (Figure S16b). This fundamental difference in the behavior between A_3_V_2_O_8_ and Ba_7_Nb_4_MoO_20_ helps explain why hydration of the former is severely limited. The hexagonal derivative Ba_3_VWO_8.5_, which is constituted by layers of isolated V/W polyhedral units spaced by ordered cationic vacancies analogous to the palmierite structure, similarly does not exhibit significant water absorption because of the strong preference of V^5+^ for tetrahedral geometry.^65^ It is likely that the as-prepared A_3_V_2_O_8_ materials present a small oxygen non-stoichiometry (not visible by neutron diffraction and generated by heating or the high temperature synthesis), which enables low concentrations of water absorption. To confirm this, we considered the possibility of water molecules absorbing at pre-existing oxygen vacancy sites by comparing the energetics of the materials with an oxygen vacancy with those of the hydrated (0.0825H_2_O) phases. This resulted in exothermic hydration enthalpies of −63.00 and −32.81 kJ mol^–1^ for Sr_3_V_2_O_8_ and Ba_3_V_2_O_8_, respectively, suggesting that the hydration of these materials can become favorable when they are sufficiently oxygen-deficient. Given that the water concentrations in the models are significantly higher than in the experimental samples, the observed enhancement in conduction observed from the simulations may represent a potential route to increased conductivity in these materials. However, this is reliant on achieving high proton concentrations in the materials experimentally, possibly through acceptor doping strategies.

**Figure 6:**
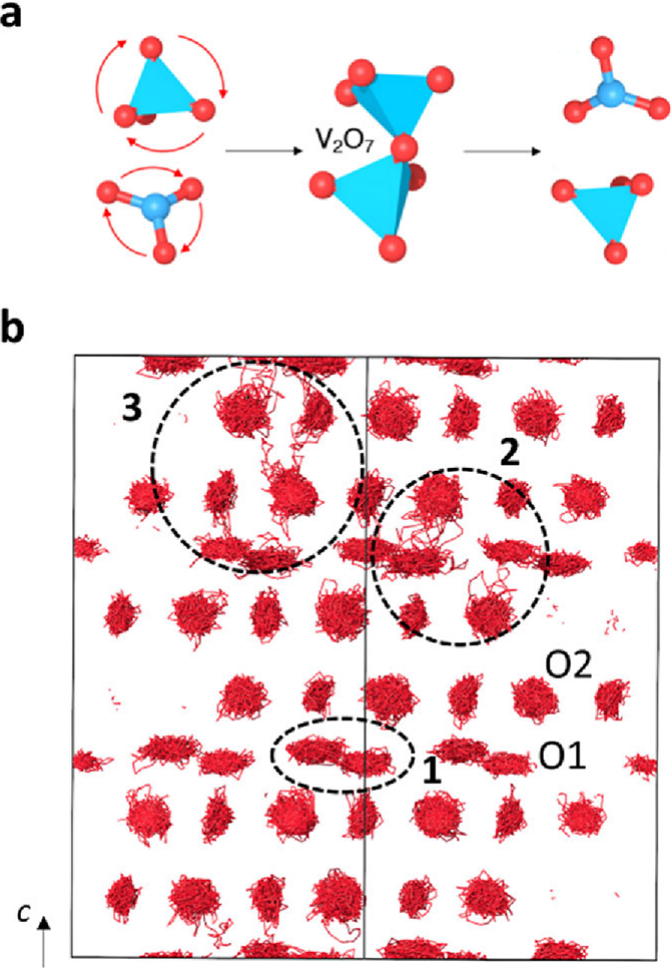
(a) Schematic of the oxygen migration mechanism via the formation of a V_2_O_7_ group via condensation of a VO_4_ tetrahedron with an oxygen-deficient VO_3_ group. Rotation of the polyhedral units facilitates the ionic transport. (b) AIMD trajectory plot for oxide ions in Sr_3_V_2_O_8_ with an oxygen vacancy concentration of 6.25% at 1200 K. The circled areas evidence exchange between O1–O1 (**1**), O1–O2 (**2**), and O2–O2 (**3**) positions.

AIMD simulations were also used to determine the mechanism of oxide ion transport in these materials. We considered two models of Sr_3_V_2_O_8_, i.e., with and without oxygen vacancies. In the pristine system without defects, no long-range ion diffusion or VO_4_ rotational disorder is observed. In contrast, in the system with an oxygen vacancy concentration of 6.25%, both long-range ion diffusion and VO_4_ rotational disorder are found, and our simulations reveal that they are unequivocally linked. As shown in Figure 6a, oxide ion transport in these materials is vacancy-driven and occurs via the formation of V_2_O_7_ groups, where an oxygen atom from a VO_4_ group transitions to the vacant site of an oxygen-deficient VO_3_ group. Given the relatively large interatomic distances between the V ions in Sr_3_V_2_O_8_ and Ba_3_V_2_O_8_ (> 3.76 and 3.95 Å, respectively), the rotation of VO_4_ groups is pivotal in facilitating ion transport in these materials. The AIMD simulations show a three-dimensional oxide-ion diffusion pathway, with exchange between O1–O1, O1–O2, and O2–O2 positions (see circled areas in Figure 6b). This is in contrast with the two-dimensional oxide ion diffusion pathway along the *ab* plane reported for hexagonal perovskite derivatives containing palmierite-like layers.^64,66^

Bond–valence site energy calculations similarly prove three-dimensional connectivity across the different oxygen positions (Figure S17). The relative BVS energy barriers for O1–O2 and O2–O2 hopping (∼ 0.30 and ∼ 0.55 eV, respectively) are comparable with the barrier for O1–O1 hopping (∼ 0.23 eV) and considerably lower than the analogous BVSE barriers for oxygen migration along the *c* axis reported for Ba_3_*M*′*M*″O_8.5_ (*M′* = V, Nb, Ge; *M″* = Mo) hexagonal derivatives (>0.70 and > 1.1 eV),^39,67^ thus demonstrating that these pathways can offer a sizable contribution to the oxide ion diffusion in A_3_V_2_O_8_, probably due to the ease of rotation of the polyhedral units. Such mechanism of oxygen migration via the synergic rotation and deformation of VO_4_ units to allow the breaking and reforming of V_2_O_7_ dimers is analogous to the one reported in La_1-*x*_Ba_1+*x*_GaO_4-*x*/2_ and scheelite Bi_1-*x*_Sr_*x*_VO_4–0.5*x*_, both formed by isolated tetrahedral moieties.^28,31^ It is likely that the high dynamical and rotational flexibility of the isolated tetrahedral moieties also assist the proton transport, in analogy with the case of Ba_7_Nb_4_MoO_20_ and the solid-acid protonic conductor CsH_2_PO_4_.^64,68^

## Conclusions

In summary, we reported significant proton and oxide ion conductivity in palmierite oxides A_3_V_2_O_8_ (A = Sr, Ba). These systems present prevalent ionic conduction with a large protonic component under humidified air (*t*_H_ ∼ 0.6–0.8). In particular, the proton conductivity of Sr_3_V_2_O_8_ is competitive with other proton conductors constituted by isolated tetrahedral units. Protons incorporated in the A_3_V_2_O_8_ structure have high mobility, and our results suggest that the introduction of extrinsic oxygen vacancies by chemical acceptor doping can favor water absorption and further increase both the proton and oxide ion conductivity. Proton and oxide ion conduction through stabilization of oxygen vacancy defects in oxide-type structures constituted by isolated tetrahedral units is rare, with only examples constituted by scheelite-type oxides and doped La_1-*x*_Ba_1+*x*_GaO_4-*x*/2_.^28,31,69^ Studies on hexagonal perovskite derivatives have evidenced that oxygen interstitial defects can also be stabilized within palmierite-like motifs, resulting in increased ionic conductivity.^70^ Palmierite oxides constitute a promising new family of ionic conductors where stabilization of parallel vacancy and interstitial defects can be employed for the design of materials with improved conductivities.

## Supporting Information Available

Figures showing further electrochemical data; details of the equivalent circuit analysis; figures and tables of crystallographic data; figures and tables of further results of DFT, AIMD, and BVSE calculations; thermogravimetric data (PDF)
