# Supplementary material for: Proton and Oxide Ion Conductivity in Palmierite Oxides
Source: Chem Mater. 2022 Sep 6;34(18):8190–7. doi: 10.1021/acs.chemmater.2c01218 (PMC9523575; doi:10.1021/acs.chemmater.2c01218)
Supplement: Supplementary file 1 — cm2c01218_si_001.pdf [file cm2c01218_si_001.pdf]

Supplementary information for

Proton and Oxide Ion Conductivity in Palmierite Oxides

Sacha Fop,[1,2,*] James A. Dawson,[3,4] Dylan N. Tawse,[2] Matthew G. Skellern,[2] Janet M. S. Skakle,[2] and Abbie C. Mclaughlin[2]

[1] ISIS Facility, Rutherford Appleton Laboratory, Harwell OX11 0QX, United Kingdom
[2] The Chemistry Department, University of Aberdeen, Aberdeen AB24 3UE, United Kingdom
[3] Chemistry – School of Natural and Environmental Science, Newcastle University, Newcastle NE1 7RU, United Kingdom
[4] Centre for Energy, Newcastle University, Newcastle NE1 7RU, United Kingdom

* sacha.fop@stfc.ac.uk

Table S1. Lattice parameters for the computed structures at 0 K. The calculated unit cell parameters are in good agreement with the experimental values (see Tables S2 and S3).

	a **(Å)**	***c*** **(Å)**
$Sr_3V_2O_8$	5.590	20.079
$Sr_3V_2O_8 \cdot 0.0825H_2O$	5.621	20.060
$Sr_3V_2O_8 \cdot 0.330\ H_2O$	5.651	20.059
$Ba_3V_2O_8$	5.762	21.357
$Ba_3V_2O_8 \cdot 0.0825H_2O$	5.777	21.355
$Ba_3V_2O_8 \cdot 0.330H_2O$	5.843	21.303

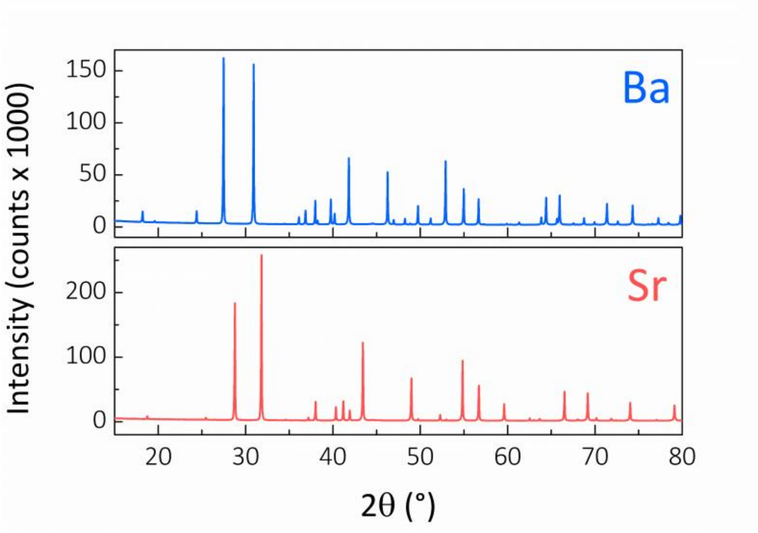

Figure S1. Laboratory X-ray diffraction patterns of as prepared $A_3V_2O_8$ (A = Sr, Ba).

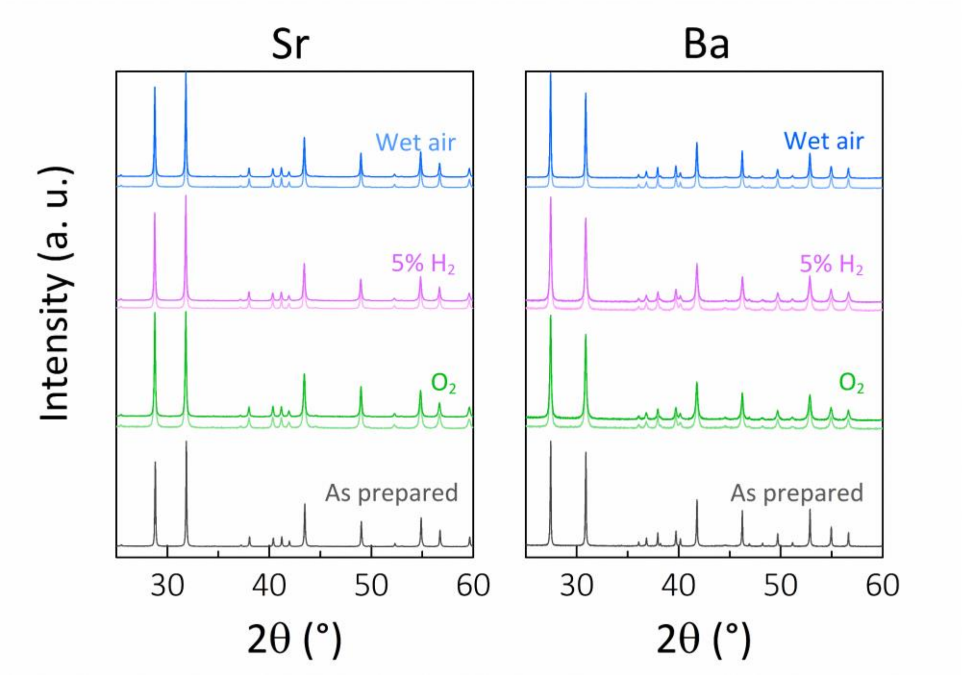

Figure S2. Laboratory X-ray diffraction patterns of $A_3V_2O_8$ (A = Sr, Ba) after annealing for 10 hours at 600 and 800 °C (bottom lighter line and top darker line, respectively) under O_2, 5% H_2/N_2 and humidified air. There is no change in the XRD patterns thus indicating the high phase stability of the as prepared materials.

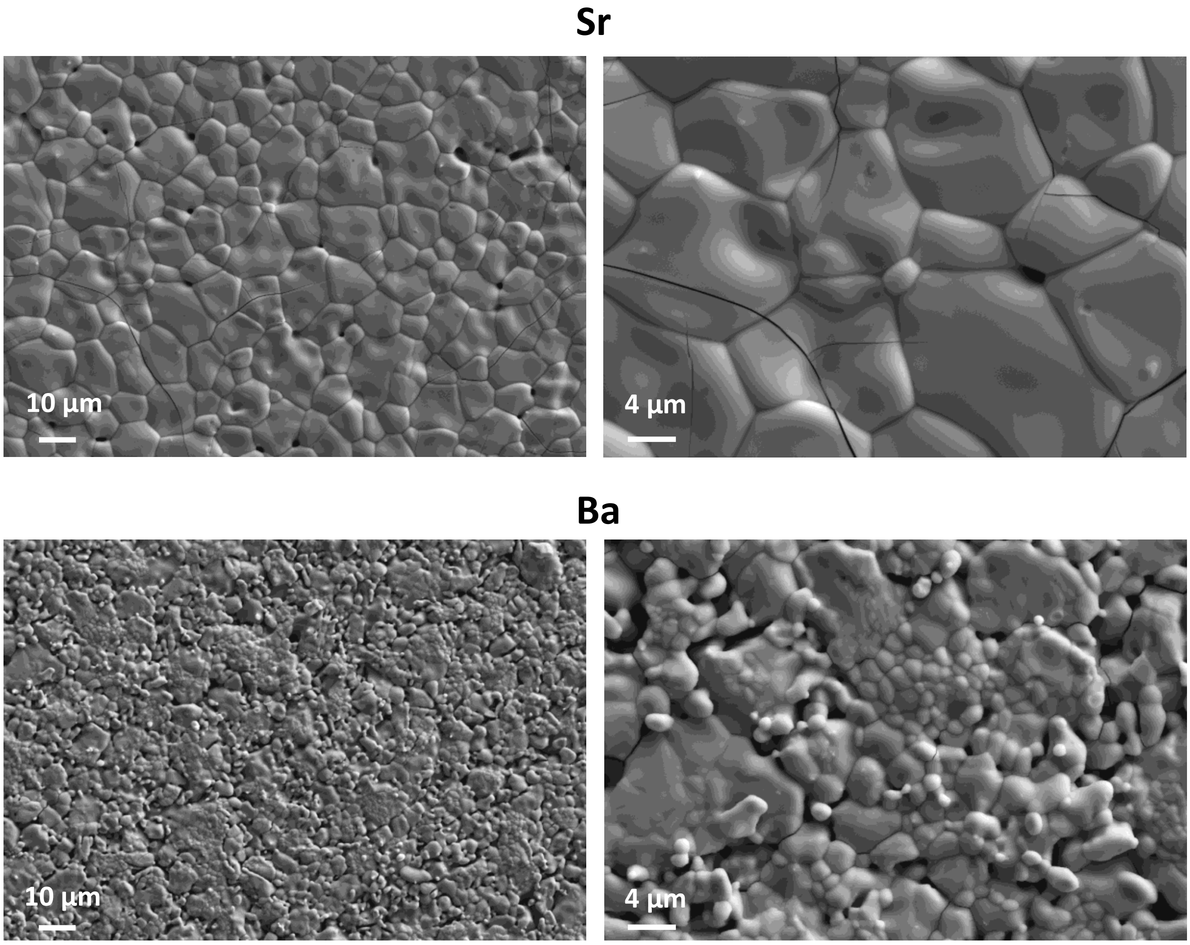

Figure S3. SEM micrographs of the surface dense $A_3V_2O_8$ (A = Sr, Ba) pellets. The images show grains ranging in size between ~ 10 – 20 µm for $Sr_3V_2O_8$ and between ~ 1 – 5 µm for $Ba_3V_2O_8$.

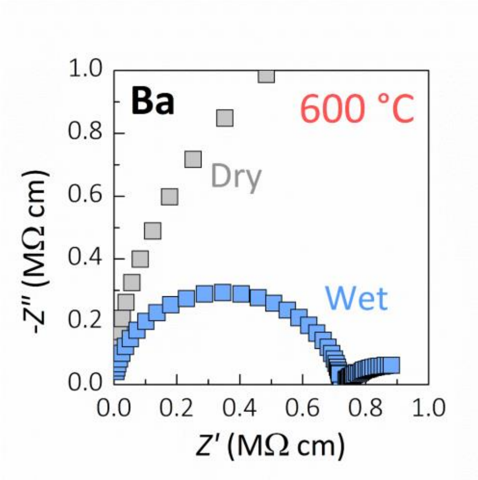

Figure S4. Complex impedance plot of $Ba_3V_2O_8$ collected under dry and wet air at 600 °C. The data show a clear reduction in resistivity on going from dry to humidified air. The complex impedance plot collected under wet air presents a Warburg response at low frequencies.

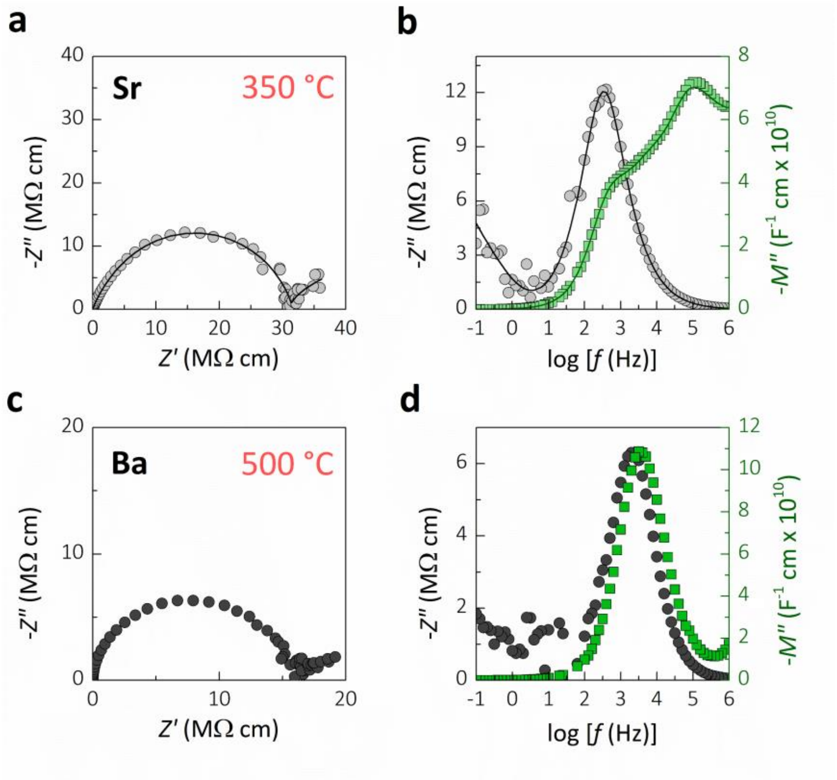

Figure S5. Complex impedance plot **(a)** and complex modulus (*M*”) and impedance (*Z*”) spectroscopic (Bode) plots **(b)** of $Sr_3V_2O_8$ collected under dry air at 350 °C. The complex modulus plot shows two peaks, one associated to the bulk response (high frequency) and the other associated to the grain boundary response (intermediate frequency). The most resistive part of the sample (grain boundary) is highlighted by the *Z*” spectroscopic plot, while the modulus plot highlights the element with the smallest capacitance (bulk) [1]. The lines represent the equivalent circuit fitting to the data. Complex impedance plot **(c)** and Bode plots **(d)** of $Ba_3V_2O_8$ collected under dry air at 500 °C. The complex modulus and impedance spectroscopic plots show a single peak which is associated to the overall grain response.

2. Equivalent circuit analysis

An equivalent circuit (EC) fitting procedure was employed to extract the individual bulk, grain boundary and electrode responses for the impedance data of $Sr_3V_2O_8$ collected under dry and humidified air atmosphere. The following equivalent circuit was used to model the impedance data,

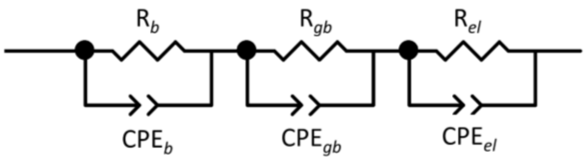

where R indicates a resistor, CPE a constant phase element; the subscript *b* stands for bulk, *gb* for grain boundary and *el* for electrode.

The complex impedance data of $Sr_3V_2O_8$ are composed by overlapping bulk and grain boundary arcs, together with a pronounced Warburg signal in the low frequency region. Equivalent circuit fits at selected temperatures are presented in Figure S4. The good match between the observed and calculated spectra is evident from the graphs.

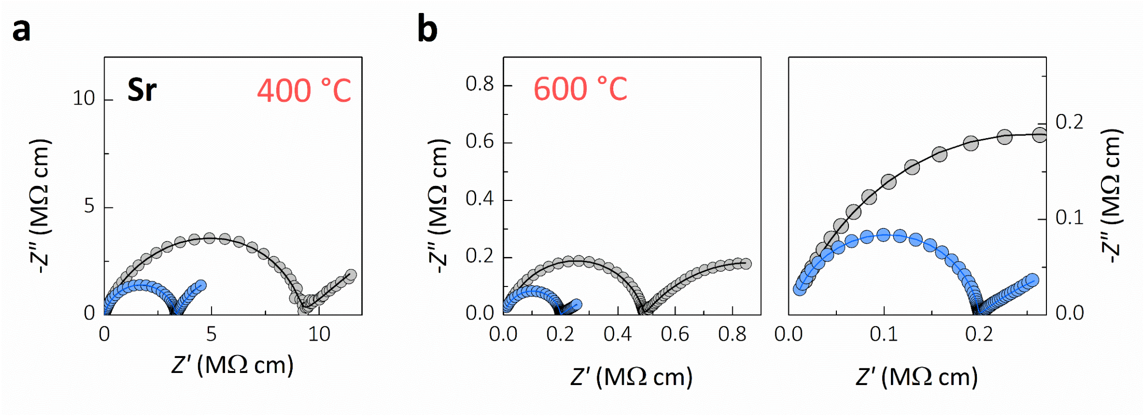

Figure S6. Complex impedance plots of $Sr_3V_2O_8$ recorded under dry (grey symbols) and humidified (blue symbols) air at 400 °C **(a)** and 600 °C **(b)**. The lines represent the equivalent circuit fitting. The right panel in **(b)** is a magnification of the wet air data.

The validity of the equivalent circuit analysis can be further evaluated by inspection of the distribution of the EC residuals ($\Delta Z'_{EC}$, $\Delta Z''_{EC}$) versus the logarithm of the frequency. The residuals from the equivalent circuit analysis present small values, with a variation generally smaller than 2% for the dry air data and smaller than 0.5% in the case of the data collected under humidified air (Figure S4). The values are also reasonably scattered along log(*f*), further validating the quality of the employed model [2].

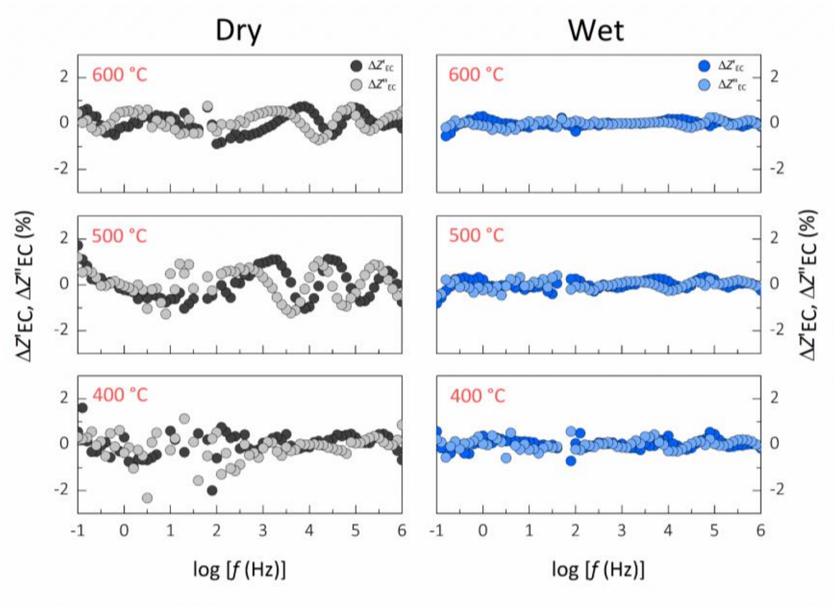

Figure S7. Typical residual plots of the equivalent circuit fitting procedure for the impedance data of $Sr_3V_2O_8$ at selected temperatures. The good match between observed and calculated impedance data, and the generally random distribution of the residuals indicate that the employed equivalent circuit model adequately represents the collected data.

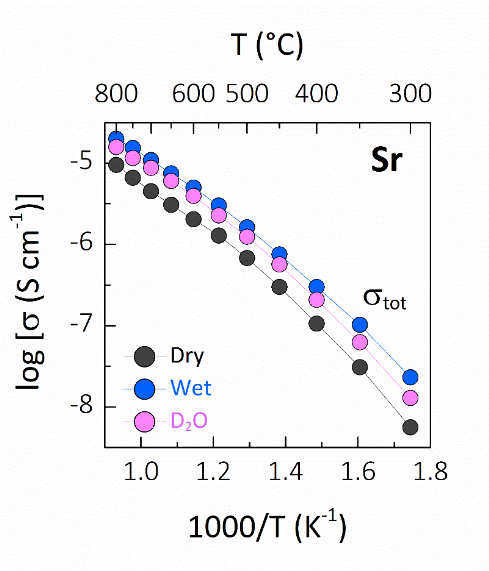

Figure S8. Arrhenius plot showing a reduction of the total conductivity of $Sr_3V_2O_8$ under air + D_2O atmosphere due to the isotope effect.

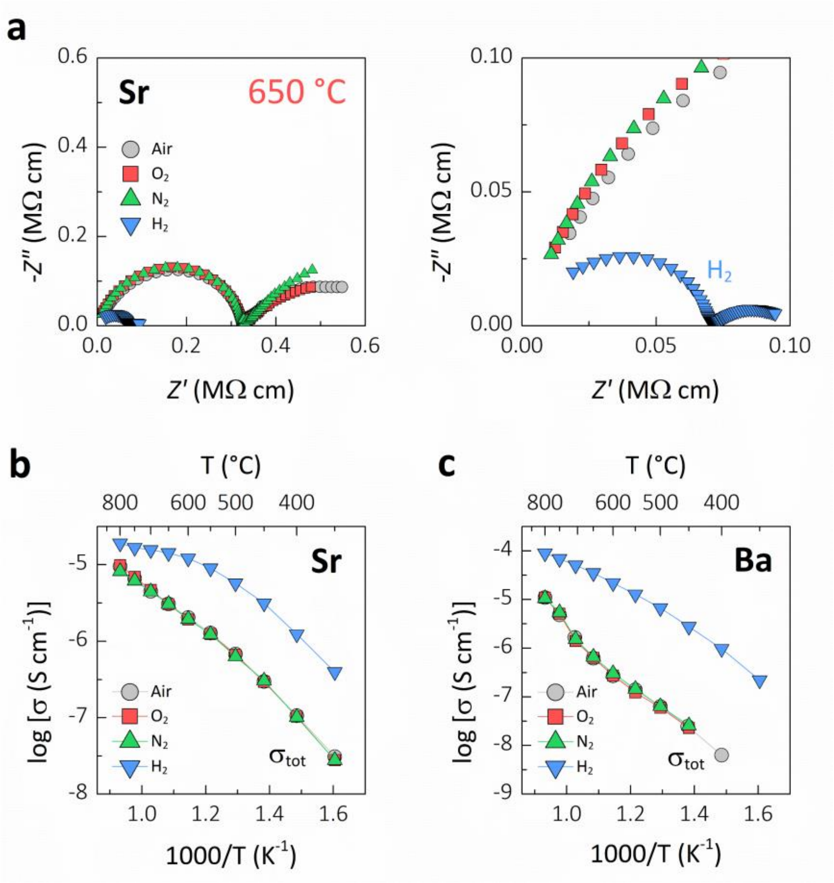

Figure S9. (a) Complex impedance plots of $Sr_3V_2O_8$ recorded under dry atmospheres (air, O_2, N_2 and 5% H_2 in N_2) at 650 °C. The right panel shows a magnification of the data collected under dry hydrogen atmosphere. The sample response is independent of the atmosphere at high-intermediate partial pressure of oxygen. The behaviour of the low-frequency electrode signal is consistent with oxygen ion conduction and Warburg diffusion [1]. **(b)**, **(c)** Arrhenius plots of the total conductivities of $Sr_3V_2O_8$ and $Ba_3V_2O_8$ under dry atmospheres. The total conductivity does not depend on the oxygen partial pressure at high and intermediate pO_2 values, demonstrating that the $A_3V_2O_8$ samples present predominantly ionic (oxide ion) conductivity under dry atmospheres. There is an increase in the conductivity in 5% H_2 in N_2 (which is particularly pronounced for $Ba_3V_2O_8$) revealing an electronic *n*-type component under the more reducing conditions.

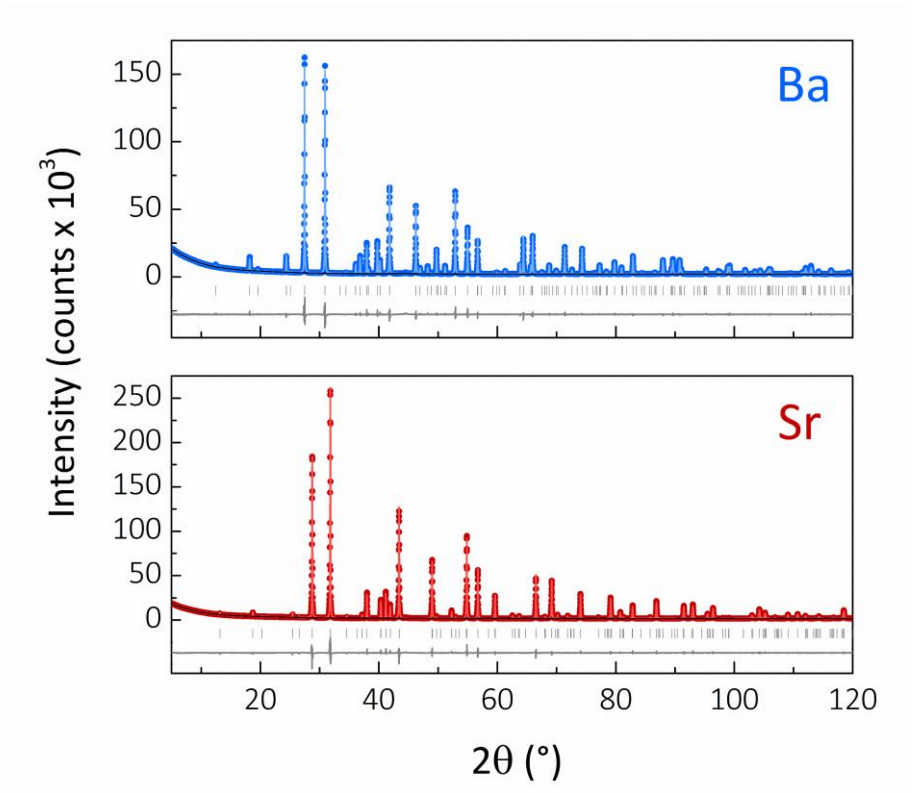

Figure S10. Rietveld histograms for the X-ray diffraction data of $A_3V_2O_8$ (A = Sr, Ba).

Table S2. Refined atomic parameters from Rietveld fit of the high-resolution X-ray diffraction data of $A_3V_2O_8$ (A = Sr, Ba) collected at room temperature (25 °C). Data were refined in the space group $R\bar{3}m$. The fractional occupancies of the different atoms refined within ± 1% of the full occupancy and were fixed to 1. All atoms were refined with isotropic displacement parameters, U_{iso} (Å^2).

			Sr	Ba
	a (Å)		5.61801(2)	5.78366(2)
	c (Å)		20.10050(9)	21.32148(9)
	V (Å^3)		549.420(5)	617.667(5)
			**Sr**	**Ba**
**A1**	**3*a* (0,0,0)**	U_{iso}	0.0098(3)	0.0118(2)
**A2**	**6*c* (0,0,*z*)**	*z*	0.202071(4)	0.205288(2)
		U_{iso}	0.0015(5)	0.0009(1)
**V1**	**6*c* (0,0,*z*)**	*z*	0.4059(2)	0.40699(9)
		U_{iso}	0.0025(5)	0.0014(2)
**O1**	**6*c* (0,0,*z*)**	*z*	0.3230(1)	0.3315(6)
		U_{iso}	0.0288(5)	0.0455(6)
**O2**	**18*h* ($x,\bar{x},z$)**	*x*	0.1698(2)	0.1541(7)
		z	0.5666(3)	0.5661(5)
		U_{iso}	0.0189(6)	0.0191(3)
			**Sr**	**Ba**
	χ^2		6.34	5.09
	**Rp (%)**		4.26	3.42
	**Rwp (%)**		6.63	5.27

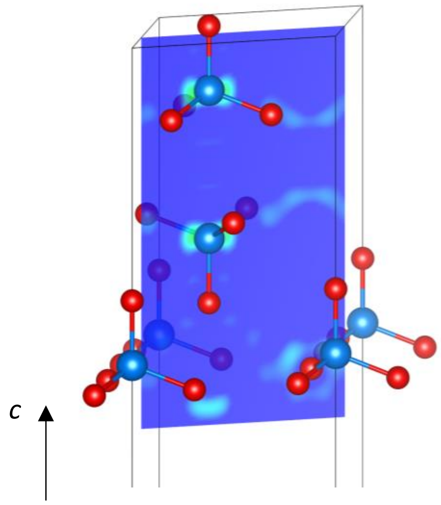

Figure S11. Section of X-ray difference Fourier map for $Sr_3V_2O_8$ as seen along the [100] direction at a y ~ 0.333. The metal V cations are represented by blue spheres, while the oxygen atoms are in red. The Ba atoms are omitted for clarity. Examination of X-ray difference Fourier maps shows no evidence of significant missing electron scattering density between the two V positions, indicating that the site is empty and the cation vacancies are fully ordered.

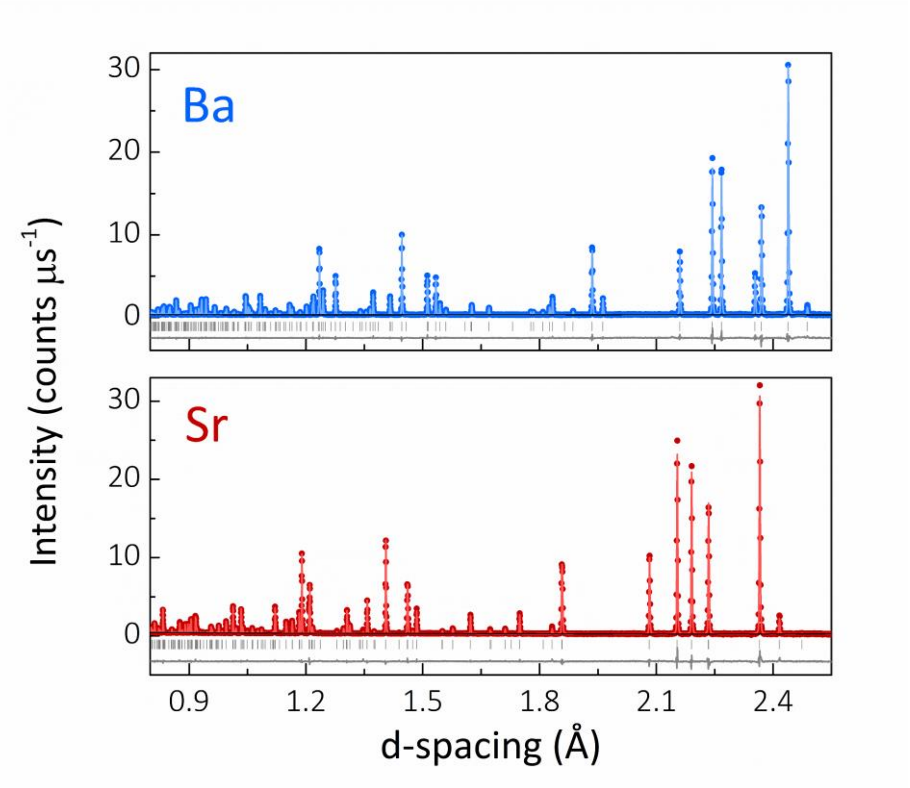

Figure S12. Rietveld histograms for the neutron diffraction data of $A_3V_2O_8$ (A = Sr, Ba) collected on HRPD. The TOF neutron diffraction data for the high-resolution backscattering detector of HRPD is shown.

Table S3. Refined atomic parameters from Rietveld fit of the high-resolution neutron diffraction data of $A_3V_2O_8$ (A = Sr, Ba) collected at room temperature (25 °C). Data were refined in the space group $R\bar{3}m$. The fractional occupancies of Ba and O refined within ± 1% of the full occupancy and were fixed to 1. The fractional occupancies of V were fixed to 1 from the results of the refinements of the structure from X-ray diffraction. The U_{13}, U_{23} anisotropic displacement parameters are zero for A1, A2, V1 and O1 due to the symmetry of the unit cell.

				Sr	Ba
	***a* (Å)**			5.61913(1)	5.784345(9)
	***c* (Å)**			20.10422(7)	21.32742(5)
	***V* (Å³)**			549.740(2)	617.984(2)
				**Sr**	**Ba**
**A1**	**3*a***	**(0,0,0)**	$U_{11} = U_{22}$	0.0092(4)	0.0159(4)
			U_{33}	0.0053(5)	0.0071(5)
			U_{12}	0.0046(2)	0.0080(2)
**A2**	**6*c***	**(0,0,*z*)**	z	0.201981(8)	0.205054(3)
			$U_{11} = U_{22}$	0.0100(3)	0.0107(3)
			U_{33}	0.0016(4)	0.0092(4)
			U_{12}	0.0050(1)	0.0054(1)
**V1**	**6*c***	**(0,0,*z*)**	z	0.4070(4)	0.4063(4)
			$U_{11} = U_{22}$	0.0132(3)	0.0068(1)
			U_{33}	0.0135(2)	0.0072(1)
			U_{12}	0.0066(2)	0.0034(2)
**O1**	**6*c***	**(0,0,*z*)**	z	0.32244(3)	0.327876(2)
			$U_{11} = U_{22}$	0.0315(4)	0.0331(3)
			U_{33}	0.0050(3)	0.0079(4)
			U_{12}	0.0157(2)	0.0166(1)
**O2**	**18*h***	**($x,\bar{x},z$)**	x	0.16762(5)	0.16144(5)
			z	0.56528(3)	0.565546(1)
			U_{11}	0.0242(2)	0.0167(2)
			U_{22}	0.0040(3)	0.0167(2)
			U_{33}	0.0082(3)	0.0164(3)
			U_{12}	0.0020(1)	0.0120(2)
			U_{13}	0.0010(1)	0.0006(1)
			U_{23}	-0.0010(1)	-0.0006(1)
				**Sr**	**Ba**
	χ^2			4.21	2.35
	**Rp (%)**			4.70	3.60
	**Rwp (%)**			4.78	3.16

Table S4. Selected bond lengths and angles for $A_3V_2O_8$ (A = Sr, Ba).

	**Sr**	**Ba**
**A1–O1** (Å)	3.25179(7)	3.34162(2)
**A1–O2** (Å)	2.6096(7)	2.7599(4)
**A2–O1** (Å)	2.4192(13)	2.6195(7)
**A2–O2** (Å)	2.8736(2)	2.9501(1)
	2.5972(8)	2.8070(7)
**V1–O1** (Å)	1.703(1)	1.673(8)
**V1–O2** (Å)	1.708(5)	1.725(3)
**O1–V1–O2** (°)	109.0(4)	110.4(2)
**O2–V1–O2** (°)	109.9(2)	108.6(2)

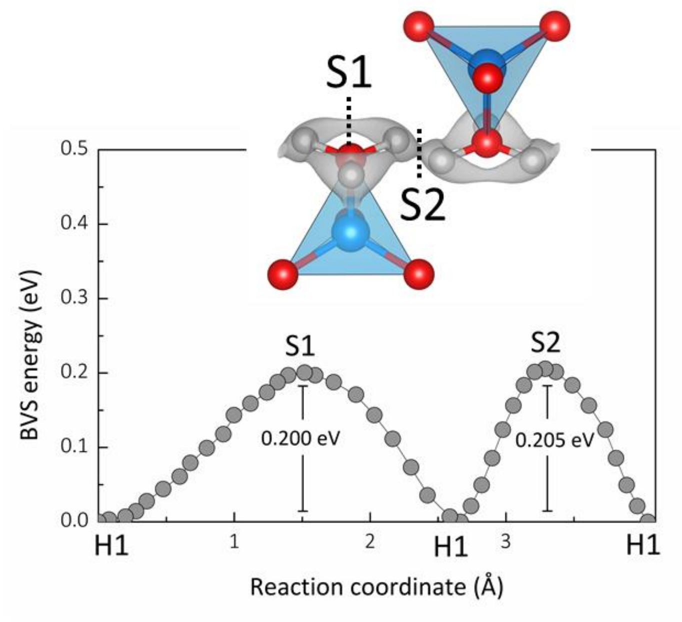

Figure S13. Connectivity between BVSE isosurfaces reveals a lowest energy 2-dimensional proton conduction pathway (isosurface levels at < 0.3 eV) composed by H1–H1 exchange and hopping onto adjacent oxygen atoms. BVSE model of proton migration barriers for proton exchange (S1) and hopping (S2) for $Sr_3V_2O_8$.

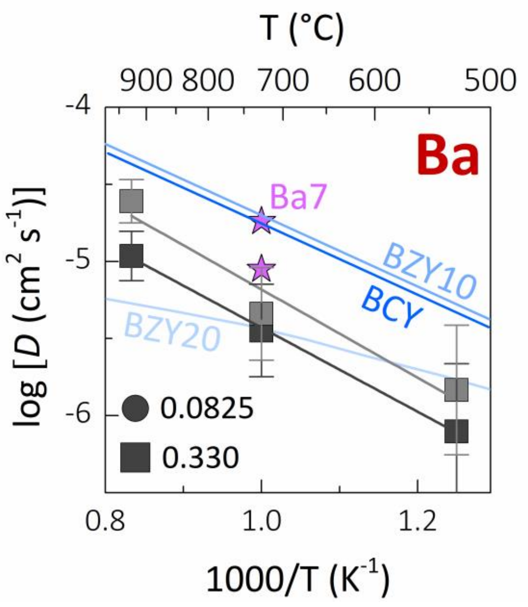

Figure S14. Arrhenius plot of the calculated proton diffusion coefficient for $Ba_3V_2O_8{\cdot}nH_2O$, with n = 0.0825 and 0.330. The diffusion coefficients for perovskite-type proton conductors $BaCe_{0.98}Y_{0.02}O_{3-\delta}$ (BCY) [3], $BaZr_{0.9}Y_{0.1}O_{3-\delta}$ (BZY10) [3] and $BaZr_{0.8}Y_{0.2}O_{3-\delta}$ (BZY20) [4], as week as for $Ba_7Nb_4MoO_{20}{\cdot}nH_2O$ with n = 0.125 and 0.5 (Ba7) [5] are also plotted for comparison. The error bars correspond to the statistical uncertainty in the fitting of the mean square displacement to time curve.

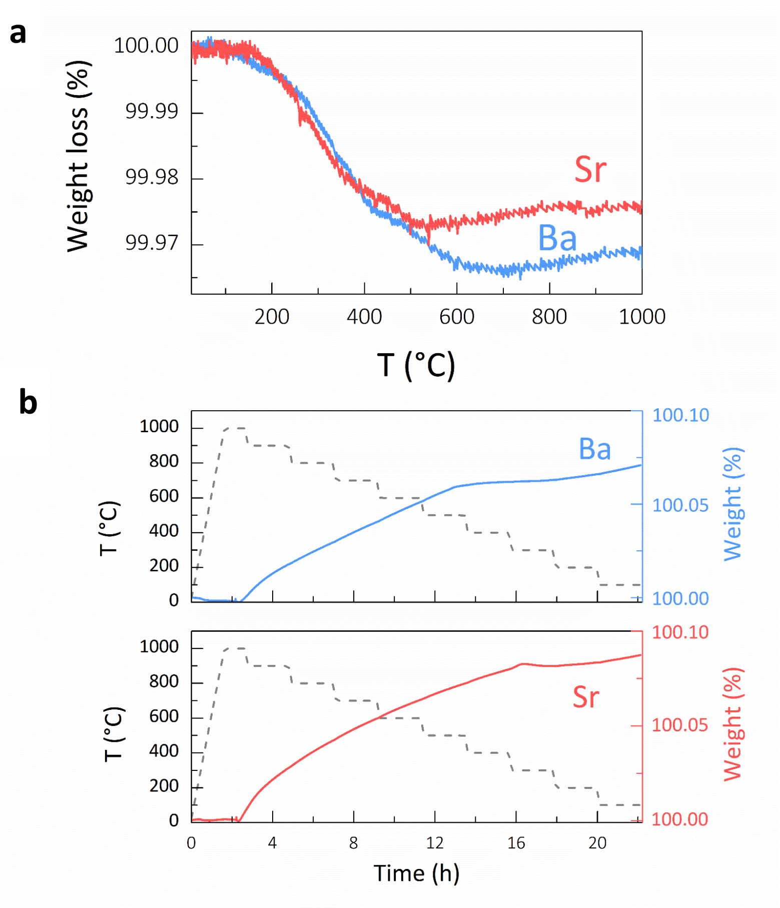

Figure S15. (a) Results of thermogravimetric analysis on $A_3V_2O_8{\cdot}nH_2O$ (A = Sr, Ba) samples stored at ambient conditions. The weight losses correspond to water concentration of n = 0.008 for A = Sr and n = 0.013 for Ba. **(b)** Water uptake of $A_3V_2O_8$ samples measured by equilibrium isotherms with thermogravimetric analysis under humidified air (pH_2O ~ 0.021 atm). The samples show water uptakes of 0.024 molecules of water per formula unit for $Sr_3V_2O_8$ and 0.026 for $Ba_3V_2O_8$. Given the small amount of water uptake, the obtained values are potentially overestimated due to buoyancy effects during the measurements.

Table S5. Hydration enthalpies of $A_3V_2O_8{\cdot}nH_2O$ (A = Sr, Ba) obtained by DFT calculations for different water uptakes (*n*). Hydration enthalpies were calculated based on the energy differences between the hydrated and dehydrated materials. For the materials with the lower water content ($0.0825H_2O$), we also considered the possibility of water molecules absorbing at pre-existing oxygen vacancy sites by comparing the energetics of the materials with an oxygen vacancy with those of the hydrated phases.

A	nH_2O		Hydration enthalpy (kJ mol^{-1})
Sr	0.0825		+10.61
	0.330		+87.80
	0.0825	Oxygen vacancy site	-63.00
Ba	0.0825		+68.51
	0.330		+77.19
	0.0825	Oxygen vacancy site	-32.81

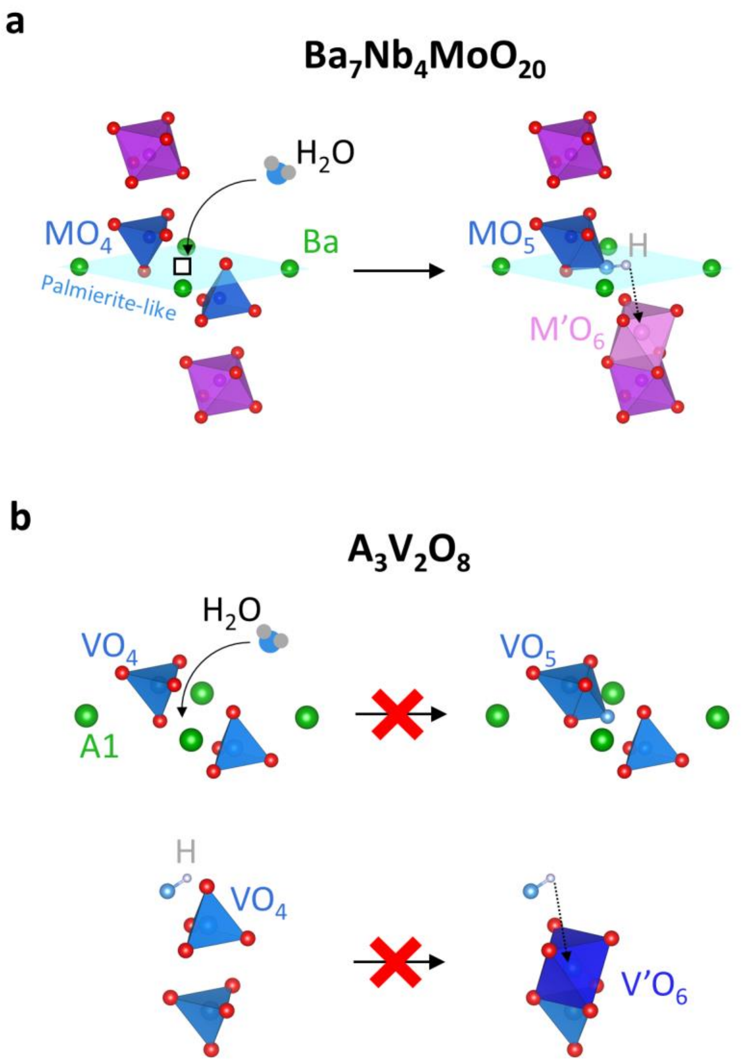

Figure S16. (a) Schematic of the water absorption in $Ba_7Nb_4MoO_{20}$. Hydration leads to change of the local metal coordination and can force the position of a metal from a tetrahedral site within the palmierite-like layer to an adjacent vacant octahedral metal site, with consequent formation of a M'O_6 unit [5]. **(b)** Contrary to $Ba_7Nb_4MoO_{20}$, results from DFT geometry optimization demonstrate that increase in coordination of the VO_4 units due to water absorption cannot occur in $A_3V_2O_8$ (top panel). The shift of a vanadium cation from the tetrahedral site to an empty adjacent octahedral site due to the repulsion of a proton does not occur either (bottom panel).

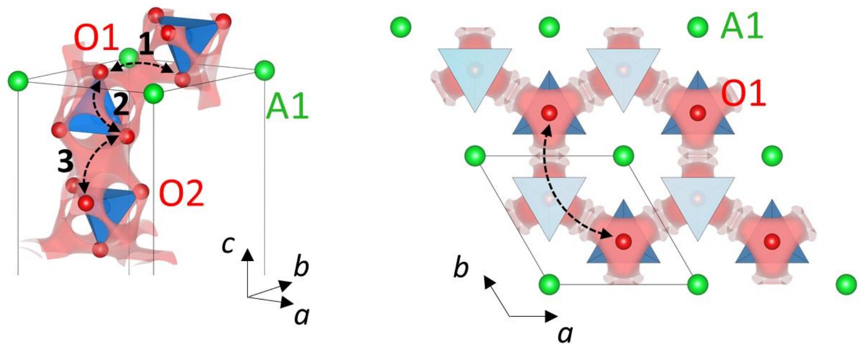

Figure S17. BVSE map for a test O^{2-} ion in $Sr_3V_2O_8$ showing three-dimensional connectivity suggesting exchange between O1–O1 (**1**), O1–O2 (**2**) and O2–O2 (**3**) positions. The panel on the left shows the BVSE path at z = 0 as seen along the [001] direction evidencing a curved trajectory around the A1 cations. Isosurfaces levels are drawn at < 0.6 eV.

References

[1] Irvine, J. T. S., Sinclair, D. C. & West, A. R. Electroceramics: Characterization by Impedance Spectroscopy. *Adv Mater* **2**, 132-138 (1990).

[2] Boukamp, B. A. Electrochemical Impedance Spectroscopy in Solid State Ionics: Recent Advances. *Solid State Ionics* **169**, 65-73 (2004).

[3] Münch, W.; Kreuer, K. -.; Seifert, G.; Maier, J. Proton Diffusion in Perovskites: Comparison Between $BaCeO_3$, $BaZrO_3$, $SrTiO_3$, and $CaTiO_3$ Using Quantum Molecular Dynamics. *Solid State Ionics* **2000***, 136-137*, 183-189.

[4] Yamazaki, Y.; Blanc, F.; Okuyama, Y.; Buannic, L.; Lucio-Vega, J.; Grey, C. P.; Haile, S. M. Proton Trapping in Yttrium-Doped Barium Zirconate. *Nature Materials* **2013***, 12*, 647-651.

[5] Fop, S.; Dawson, J. A.; Fortes, A. D.; Ritter, C.; McLaughlin, A. C. Hydration and Ionic Conduction Mechanisms of Hexagonal Perovskite Derivatives. *Chem. Mater.* **2021***, 33*, 4651-4660.
